# Immoral Advertisements

**Published:** 1858-02

**Authors:** 


### Immoral Advertisements.

We have for a length of time thought of directing attention to a species of advertising, to be found in many of the newspapers of the day, which we can not regard in any other light than as grossly *criminal* upon the part of the authors, and in the highest degree censurable upon the part of those who control the press, which should be a vehicle for the lessons of morality and virtue, rather than those of vice and infamy. We have no idea that we shall accomplish much by what we say upon this subject; but we are determined to raise our feeble voice against what we can not possibly regard in any other light than as the gravest offence against the well-being of Society.

In this connection, we take pleasure in referring to and quoting an editorial article from the "Cincinnati Lancet and Observer," which expresses in a most forcible manner our views upon this subject. The Editors remark, under the head of "Abortion Pills:"—

"We have for some time felt a disposition to enter our protest against a villanous form of advertising, which defiles the columns of a very large portion of our secular newspapers: we allude of course to "Female Pills," which are freely advertised under a variety of names, as 'Sir James Clark's celebrated Female Pills,' and with other titles—all, however, with this saving clause, by way of caution! that these pills are not to be taken by females while in a pregnant condition, as they are sure to bring on miscarriage; of course nobody is mistaken in the design of these cautions or the purpose for which those remedies are thus paraded in all our family newspapers throughout the land. They are a grand rascality, and the author of these remedies and advertisements are worthy of a high place in our State Prisons—public opinion and public contempt will not reach such knaves. We are sorry also to see that the publishers of our most respectable newspapers so readily make themselves *particeps criminis* in this matter; we certainly think they have not reflected upon the *nature* and *tendency* of these advertisements, which thus thrust into the hands of our sisters and daughters all over the country—in almost so many words, whisper the insidious language of the tempter in their ears—'thou shalt not surely die.'

"Several of our cotemporaries have already justly censured these publications. Our friend of the Ohio Medical and Surgical Journal has recently shown this thing up in its true deformity, and as he very truly says, 'the most stupid female in the land who meditates wrong conduct, will recognize at once in looking over such statements, that there is a medicine that will produce *abortion.*' We have good reason to suppose that, in several instances, death has been produced through the use of these Pills, and we doubt their efficacy in any case; nevertheless, their moral character is the same, and the same in their temptation—they are essentially advertisements of *abortion medicine.* The truth is, a large portion of the community is becoming fearfully indifferent, and loose in their views of this subject, and it is high time wiser counsels were presented. *Physicians* may do good by presenting this matter in its true light to editors and publishers of newspapers. For years the venerable Mussey has refused to patronize any religious paper which advertised nostrums or Quack Medicines. Let us put a like rule in practice with the secular newspapers which are admitted to our firesides—let us politely *discontinue* such as cater to this immorality."

And, in conclusion, we take special pleasure in referring to the announcement of a secular paper in our own city, being, so far as we know, the only newspaper in the State that has eschewed this profitable, (in a pecuniary point of view,) but iniquitous patronage.

In the *Atlanta National American*, of October 14th, will be found the following editorial, under the head of "Objectionable Advertisements:"

"Objectionable Advertisements.—We frequently receive orders from philanthropic persons at the North to advertise books said to be written and published expressly "with a view to ameliorate the many hardships females are compelled to suffer in the capacity of mothers," and pills and other nostrums, which "never fail to effect a speedy and certain cure, when the instructions around the box are strictly followed."

"We believe *money-making* to be the chief object of the persons engaged in the manufacture and sale of such articles; that the articles themselves have a most pernicious tendency on so-ciety, and that benefitting the sex by them is a very remote contingency. For these reasons, we have always declined the publication of their advertisements, and we take this occasion to say to all persons engaged in the manufacture and sale of them that we will not publish such advertisements at *any price.*

"We confess ourselves to be anxious for advertising patronage, and we are well aware that this is of a class which can afford to and does pay well, but we will not consent to make our paper the vehicle for conveying into our own families, or the families of our neighbors, such information as these advertisements contain. If we cannot sustain our paper without admitting them, we prefer that it should die. It is our ardent desire to make the *National American* a paper which no head of a family shall hesitate to introduce into the family circle for fear of the demoralizing influence of even an advertisement. Our readers, we hope, have observed, also, that all tales and incidents of an offensive or even doubtful tendency, which have their origin in large towns and cities, and other populous localities, are scrupulously excluded from our columns.

"We have an abiding confidence in the correctness of our views. We shall stand or fall by them. If we lose temporarily, we feel sure of an ultimate and permanent success—as we are assured the public will sooner or later appreciate our course.

"In conclusion, we would say to *subscribers* and *readers* that you thus have a guarantee of *properly selected reading matter*, and that our advertisements will be such as you will do well to read instead of passing them over—as you are sure to be gainers instead of losers; and to *Merchants* and *others* who desire a good, reliable and substantial class of customers, we would remark, that the plan we propose to ourselves in respect to Advertisements, will secure to all who read our journal as a medium for advertising, just that class of customers."

For which we return the Editor our thanks, not only as a medical man, but as a deeply interested observer and advocate of all the means calculated to purify and elevate Society, and an opponent of all the instrumentalities for the propagation of vice and impurity; and, at the same time, would desire to commend it to the consideration of his brethren of the press throughout the country.

